# Patterns of reduced nipple aspirate fluid production and ductal lavage cellularity in women at high risk for breast cancer

**DOI:** 10.1186/bcr1335

**Published:** 2005-10-24

**Authors:** Susan A Higgins, Ellen T Matloff, David L Rimm, James Dziura, Bruce G Haffty, Bonnie L King

**Affiliations:** 1 Department of Therapeutic Radiology, Yale University School of Medicine, New Haven, CT, USA; 2 Yale Cancer Center Genetics Counseling Shared Resource, Department of Genetics, Yale University School of Medicine, New Haven, CT, USA; 3 Department of Pathology, Yale University School of Medicine, New Haven, CT, USA; 4 General Clinical Research Center, Department of Pediatrics, Yale University School of Medicine, New Haven, CT, USA; 5 Department of Radiation Oncology, UMDNJ-Robert Wood Johnson Medical School, New Brunswick, NJ, USA; 6 Department of Biological Sciences, Quinnipiac University, Hamden, CT, USA

## Abstract

**Introduction:**

Nipple aspiration is a noninvasive technique for obtaining breast fluids from the duct openings of the nipple for the evaluation of abnormalities associated with breast cancer. Nipple aspirate fluid (NAF) can be elicited from 48 to 94% of healthy women, and its production has been linked to an increased relative risk for breast cancer development. NAF production has been used in studies to guide the selection of ducts for ductal lavage, a procedure in which ducts are cannulated and flushed with saline to collect cells. In a previous multicenter trial to evaluate intraductal approaches in women at high-risk for breast cancer, NAF production was observed in 84% of the subjects. However, we observed a significantly lower proportion of fluid-yielding subjects in a similar series of high-risk women. The purpose of the present study was to identify variables associated with this reduction.

**Method:**

Nipple aspiration was performed on 33 high-risk women (defined as having a 5-year Gail model index of more than 1.7, a personal or family history of breast cancer, and/or a *BRCA1* or *BRCA2* germline mutation) to identify ductal orifices for lavage procedures. Lavage was performed on all fluid-yielding ducts and on nine non-fluid-yielding ducts.

**Results:**

Fluid-yielding ducts were identified in 12 of 33 (36%) of the subjects in the present series, compared with 16 of 19 (84%) of the subjects undergoing identical procedures at our facility during a multicenter trial (*P *= 0.001). Reduced NAF yields were associated with postmenopausal status (*P *= 0.02), BRCA germline mutations (*P *= 0.004), and risk reduction therapies, including bilateral salpingo-oophorectomy (BSO) and/or selective estrogen receptor modulators (SERMs; *P *= 0.009). All nine (100%) of the ductal lavage specimens collected from non-fluidyielding ducts were acellular, in comparison with 3 of 13 specimens from fluid-yielding ducts (*P *< .001).

**Conclusion:**

Analysis of high-risk women in the present series revealed patterns of reduced NAF production and ductal lavage cellularity compared with a previous multicenter trial. The present series included more *BRCA*-positive women, many of whom had undergone BSO and/or were using SERMs. Our data suggest that endocrine mechanisms associated with these risk-reducing therapies may be related to patterns of diminished breast fluid production.

## Introduction

Nipple aspiration is a noninvasive suction technique used to obtain breast fluid from the duct openings at the nipple. The procedure elicits droplet formation from one or more ducts in 48 to 94% of healthy, nonlactating women, and these fluids often contain exfoliated ductal epithelial cells [1-10]. The nipple aspiration technique has been studied for several decades as a potential screening tool to detect cytologic abnormalities associated with proliferative breast lesions [1,5,7,11-13]. Long-term follow-up of women undergoing nipple aspiration revealed that the risk of developing breast cancer was twofold to fivefold greater for women diagnosed with cytologic atypia relative to those who did not yield any fluid [11-13]. Interestingly, these studies revealed a continuum of relative risk that was lowest for women who did not yield any fluid, higher for women yielding acellular fluid, higher still for fluid-yielders with normal or benign cells, and highest for fluid-yielders diagnosed with atypia. Thus, the mere elicitation of acellular nipple aspirate fluid (NAF) was associated with increased relative risk.

More recently the ductal lavage technique, which employs a microcatheter to cannulate an individual duct opening and flush the associated ductal system with saline, has been used to collect higher numbers of exfoliated cells. In a multicenter trial that we participated in to evaluate this approach, nipple aspiration was performed on all subjects for the purpose of locating fluid-yielding ducts (presumed to contain higher numbers of cells) before the lavage procedure [3]. Fluid-yielding ducts were identified in 84% of the subjects overall, and in 84% of the trial subjects enrolled at our center. However, this rate fell markedly to 36% (*P *= 0.001) when we performed the procedure on a similar series of 33 high-risk women after the conclusion of the trial. Here we present data suggesting that the reduced NAF yields observed in the second series of subjects may be related to the use of risk reduction therapies including selective estrogen receptor modulators (SERMs; tamoxifen and raloxifene) and/or oophorectomy.

## Materials and methods

### Participants

Subject participation for this study was approved by the Yale Human Investigation Committee (protocol no. 11401), and informed consent was obtained in all cases. Thirty-three high-risk women (defined as women with a personal or family history of breast cancer, a 5-year Gail model index of more than 1.7, and/or a germline mutation in the breast cancer predisposition gene *BRCA1* or *BRCA2*), who were asymptomatic at enrollment, were recruited through the Yale Cancer Center Genetics Counseling Shared Resource and through referrals from several regional breast clinical practices. The enrollment criteria were identical to those of the multicenter trial that we participated in, with the exception that women taking SERMs were admitted to the present study, whereas they had been excluded from the multicenter trial. To expand our analysis of certain variables, we contacted all 19 of the multicenter trial subjects who had participated at our center by letter to request oophorectomy history, which had not been collected at the time of the trial. We received responses from 10 subjects, all of whom are included in a subset of the analyses in this report.

### Nipple aspiration and ductal lavage

Each nipple was cleansed with Nuprep exfoliant (DO Weaver & Co., Aurora, CO), and then subjected to nipple aspiration using a FirstCyte™ Aspirator (Cytyc Corporation, Boxborough, MA) to identify the location of fluid-producing duct orifices. A subject was classified as a fluid yielder if one or more drops of fluid were obtained from one or more ducts. Each productive duct was cannulated with a FirstCyte™ DL Catheter (Cytyc Corporation) and lavaged with 10 to 20 ml of sterile saline (0.9% sodium chloride, Inj., USP; Abbott Laboratories, North Chicago, IL). In addition, nine non-fluid-yielding ducts were cannulated and lavaged.

### Cytology

Cytomorphologic evaluation was performed by a board-certified cytopathologist (DLR) specifically trained in ductal lavage interpretation. Specifically, lavage samples were graded on a four-point scale on the basis of the epithelial cell morphology (benign, mildly atypical, markedly atypical, and malignant). Specimens lacking sufficient epithelial cells for classification were categorized as ICMD (insufficient cells to make a diagnosis).

### Statistics

Frequencies were compared using the two-tailed Fisher exact test and the χ^2 ^test. The proportions of fluid-yielding women were compared as follows: first, between the multicenter trial and the present series of 33 subjects; second, between subsets of the 33 subjects in the present series; and third, between subsets of the pooled group of 43 subjects.

## Results

In the present series, the overall proportion of women with fluid-yielding ducts was 12 of 33 (36%), compared with 427 of 507 (84%) for the multicenter trial at large, and 16 of 19 (84%) for the Yale trial site (*P *= 0.001; Table 1). The women in the present series underwent identical procedures administered at the same facility as the subjects who had participated at the Yale trial site. Table 2 compares the characteristics of the 33 participants enrolled in the present study with those of the multicenter trial. The subjects were similar in most respects, with the exception of *BRCA1/2* germline mutation and risk reduction therapy status. In the multicenter trial, fewer than 1% of the women were *BRCA*-positive, whereas in the present trial 16 of 33 (48.5%) of the subjects had *BRCA* germline mutations (*P *< 0.001). Another difference is that the multicenter trial excluded women who were taking SERMs, whereas in the current study, 12 of 33 (36.4%) of the participants were taking SERMs (tamoxifen or raloxifene; *P *< 0.001). There was also a higher, but statistically insignificant, proportion of postmenopausal women in the present series (*P *< 0.10). A substantial number of these women (7 of 23) had undergone surgically induced menopause before the age of 49 years as the result of bilateral salpingo-oophorectomy (BSO). The proportion of women in the multicenter trial who had undergone oophorectomy was not documented. Two of the 10 trial participants who responded to our letters reported a history of oophorectomy at the time of trial enrollment. Five women in the present series were employing both forms of risk reduction at the time of study enrollment.

Within the present series of 33 subjects, NAF production varied according to menopausal status, *BRCA* mutation status, and risk reduction status (Table 1). NAF was elicited in 5 of 23 (22%) postmenopausal versus 7 of 10 (70%) premenopausal women (*P *= 0.02), and in 11 of 17 (65%) *BRCA*-negative women versus 2 of 16 (13%) of *BRCA*-positive women (*P *= 0.004). Fluid-yielding ducts were identified in 8 of 11 (73%) women not employing any form of risk reduction, versus 5 of 22 (23%) women who had undergone oophorectomy and/or were taking SERMs at the time of enrollment into the study (*P *= 0.009). To study the independent contributions of these variables to NAF reduction, we performed a set of analyses on a pooled set of subjects including the 33 subjects from the present series plus a subset of the multicenter trial subjects for whom we had retrospectively obtained oophorectomy status. To separate the contributions of age, menopause and surgically induced menopause resulting from oophorectomy, we first stratified the 43 women into two groups: those aged 49 years and below, and those aged 50 years and above. NAF production within each group was then analyzed as a function of *BRCA* mutation or risk reduction therapy status. These comparisons are shown in Table 3 and reveal a significantly lower proportion of fluid-yielders in association with *BRCA* germline mutations (*P *= 0.008) and risk reduction strategies (*P *= 0.0003) among women below the age of 50 years. When *BRCA*-negative women above and below the age of 50 years were considered separately, there were significantly lower proportions of fluid-yielders among the women on risk reduction therapy (*P *= 0.03, *P *= 0.045). No significant association was observed between NAF production and previous breast cancer history (*P *= 0.468).

In the multicenter ductal lavage trial [3] only NAF-yielding ducts were lavaged, in accordance with the rationale that productive ducts would be more likely to contain large numbers of cells. In this study the same protocol was followed, but lavage was also performed on nine subjects who did not yield fluid; all nine of these lavage specimens were classified as ICMD. Of the 22 lavage specimens collected in this series, 4 of 22 (18%) were classified as mildy atypical, 5 of 22 (23%) as benign, and 13 of 22 (54%) as ICMD. ICMD was observed in nine of nine non-fluid-yielding ducts versus 3 of 13 (59%) fluid-yielding ducts (*P *= 0.00046). The proportion of ICMD in the multicenter trial was 84 of 383 (22%) [3].

## Discussion

The overall percentage of fluid-yielding subjects in the present series (36%) was relatively low in comparison with those reported in other studies (48 to 94%) [1-3,5-10,14] and at our site during the multicenter trial (84%) [3]. Reduced NAF production has consistently been associated with postmenopausal status and/or increasing age in other studies [1,3,10,15,16]. In the multicenter trial, fluid-yielding ducts were observed in 90% of the premenopausal women, compared with 78% of the postmenopausal women (*P *< 0.001) [3]. In the present study, NAF-yielding ducts were identified in only 22% of the postmenopausal women. The marked difference between these similar high-risk postmenopausal groups (*P *< 0.001) suggests that the overall decline observed in the present series is associated with additional variables. The subject population in the present study differed in having more women with *BRCA* germline mutations, many of whom had undergone BSO and/or were using SERMs. Seven of 23 postmenopausal women in this series were less than 49 years old and had undergone surgically induced menopause as the result of BSO. When NAF production was analyzed among the 33 subjects in relation to these variables, we found it to be significantly reduced in association with *BRCA* mutations and risk reduction therapies (Table 3). Additional analyses revealed lower NAF production in association with risk-reducing therapies in *BRCA*-negative women (*P *= 0.045) and a similar, but insignificant, trend for *BRCA*-positive women.

Although NAF and ductal lavage have been evaluated in women at high risk for breast cancer development, there have been only a handful of studies describing the application of intraductal approaches to high-risk women with *BRCA* germline mutations [17,18]. The largest study, conducted by Kurian and colleagues [18], included 75 women with high inherited risk, 56 of whom had *BRCA1/2* mutations. The overall NAF rate in this study was 48%. Although no direct correlation between fluid production and risk reduction therapies was observed, there were significantly fewer fluid-yielding subjects among women with a previous history of breast or ovarian cancer. The authors suggested therapy-related anti-hormonal mechanisms including chemotherapy-induced amenorrhea as possible explanations for the trend. Mitchell and colleagues, who performed intraductal approaches on a series of 52 women with germline *BRCA1/2* mutations, observed NAF production in 60% of all subjects (Mitchell G personal communication). Although no correlation between NAF production and previous cancer was noted in this study, significantly reduced levels of NAF secretion and ductal lavage cellularity were seen in association with natural and surgically induced menopause. Although we cannulated and lavaged only nine dry ducts, all of them were also characterized as acellular. Kurian and colleagues [18] also reported higher rates of ICMD in association with previous breast or ovarian cancer, chemotherapy, and SERM usage.

The risk-reducing benefits of SERMs and oophorectomy for breast cancer patients and women at high risk for breast cancer are well documented. Clinical trials have demonstrated that tamoxifen significantly decreases recurrence rates in breast cancer patients and markedly reduces the incidence of invasive and noninvasive breast cancer development in high-risk women [19-26]. Breast cancer risk reduction has also been observed in postmenopausal women undergoing treatment with raloxifene for osteoporosis [27]. Similarly, ovarian ablation has been shown to reduce recurrence and mortality in breast cancer patients [23,24,28,29], and prophylactic oophorectomy has been shown to significantly reduce breast cancer incidence in high-risk women, including *BRCA1* and *BRCA2* mutation carriers [30-33]. Our series included eight *BRCA1* mutation carriers, and although most *BRCA1* tumors are estrogen receptor negative, both oophorectomy and tamoxifen have been shown to protect against the development or recurrence of breast cancer in this group [24,25,30,32,33].

The biologic regulation and significance of breast duct fluid production in nonlactating women are not currently understood. Our data showing that risk reduction therapies are associated with diminished breast fluid secretion complement previous epidemiologic studies showing that NAF production is linked to increased relative risk for breast cancer development [11-13]. The fact that NAF is reduced in association with therapies that eliminate or block ovarian steroids suggests that the relationship between risk and breast fluid production is related to hormones. Although SERMs modulate the actions of estrogens, we did not analyze the relationship of NAF to SERMs and BSO separately. We therefore cannot speculate on the relative contributions of estrogens and progesterone to the observed effect. However, earlier studies did not find associations between serum estrogen levels and NAF production [9,10,34]. One of these studies reported an association between NAF production and breast fluid prolactin levels, but not estrogen levels [34]. It is possible that the relationship between NAF production and ovarian hormones involves indirect systemic and/or paracrine mechanisms.

Because NAF production and cytologic atypia are both associated with increased relative risk for breast cancer development, it is tempting to speculate that NAFs reflect the secretory activity of proliferative ductal epithelial cells. However, several recent studies have generated paradoxical results concerning the relationship between breast fluid, cytologic atypia, and breast cancer risk. These studies have revealed the presence of cytologic atypia in non-fluid-yielding ducts and in NAF-negative women [9,17,18,35]. Although seemingly at odds with the body of data on NAF, the atypical epithelial cells associated with non-fluid-yielding ducts may constitute biologically distinct variants and may hold clues for stratifying the prognostic significance of atypia. Our data suggest that reduced NAF production reflects lower endogenous hormone levels and therefore that atypical cells proliferating in non-fluid yielding ducts may represent hormone-independent variants. Kurian and colleagues suggested the possibility that women with atypia in non-fluid-yielding ducts might be at higher risk for developing estrogen-receptor-negative tumors [18].

## Conclusion

An analysis of high-risk women in the present series revealed patterns of reduced NAF production and ductal lavage cellularity in comparison with a previous multicenter trial. The present series included more *BRCA*-positive women, many of whom had undergone BSO and/or were using SERMs. Our data suggest that endocrine mechanisms associated with these risk-reducing therapies may be related to patterns of diminished breast fluid production. Although NAF production does not seem to be obligatorily linked to cytologic atypia, it may be a general indicator of endogenous reproductive hormone levels that are widely implicated in breast cancer development. More importantly, NAF seems to reflect the impact of the systemic hormonal milieu on the breast tissue itself. Thus, its production during the NAF procedure could potentially provide additional information to a woman's risk profile, and could serve as a valuable endpoint in chemoprevention trials.

## Abbreviations

*BRCA* = breast cancer predisposition gene; BSO = bilateral salpingo-oophorectomy; ICMD = insufficient cells to make a diagnosis; NAF = nipple aspirate fluid; SERM = selective estrogen receptor modulator.

## Competing interests

The authors declare that they have no competing interests.

## Authors' contributions

SH and BH performed the NAF and ductal lavage procedures. EM recruited subjects and performed informed consent. DR provided cytologic evaluation. JD performed statistical analyses. BK designed and coordinated the study, performed statistical analyses, and wrote the manuscript. All authors read and approved the final manuscript.

## Figures and Tables

**Table 1:** Proportion of subjects with ducts yielding nipple aspirate fluid in multicenter trial and present study

Group	NAF^+ ^women (%)		*P*
		
	Multicenter trial (*n *= 507)	Post-trial series (*n *= 33)	
Overall	427/507 (84)		
Yale trial site	16/19 (84)	12/33 (36)	0.001
			
Premenopausal	204/227 (90)	7/10 (70)	
Postmenopausal	209/268 (78)	5/23 (22)	<0.001
	*P *< 0.001	*P *= 0.02	
No risk reduction	ND	8/11 (73)	
Risk reduction	ND	5/22 (23)	
		*P *= 0.009	
*BRCA*^-^	ND	11/17 (65)	
*BRCA*^+^	ND	2/16 (13)	
		*P *= 0. 004	

The *P *values in the right hand column reflect comparisons between the multicenter trial and the present series. The *P *values listed in the two center columns reflect comparisons between the two groups listed immediately above them. NAF, nipple aspirate fluid; ND, not done.

**Table 2:** Comparison between characteristics of women enrolled in the multicenter trial and the present study

Characteristic	Average, number, or % (range or proportion)		*P*
		
	Multicenter trial	Post-trial series	
Age (years)	51.9 (26–81)	48.4 (31–67)	
Age at menarche (years)	12.6 (7–25)	12.7 (9–17)	
Age at first birth (years)	25.3 (14–42)	28.4 (18–39)	
Menopausal status	Unknown for 2%		
Premenopausal	45 (227/507)	30.3 (10/33)	
Postmenopausal	53 (268/507)	69.7 (23/33)	
1st-degree relatives with breast cancer	0.5 (0–5)	0.74 (0–3)	
No. of previous biopsies	1.7 (0–18)	1.1 (0–4)	
Women with previous atypia (%)	19 (96/507)	10 (3/30)	
Women with previous breast cancer (%)	57 (291/507)	45 (15/33)	
*BRCA*^+ ^women (%)	<1 (3/507)	48.5 (16/33)	<0.001
Women taking SERMs (%)	0	36.4 (12/33)	<0.001
Women with oophorectomy (%)	Unknown^a^	51.5 (17/33)	
Racial heritage			
White	84	100	
African-American	12	0	
Asian	2	0	
Other	2	0	

^a^Oophorectomy history was not recorded during the trial, but was determined retrospectively for 10 of 19 subjects who participated at our site.

*P *values reflecting insignificant associations are not shown. SERM, selective estrogen receptor modulator.

**Table 3:** Proportions of women with fluid-yielding ducts within the pooled group of 43 subjects

Subjects	NAF^+ ^women		*P*
		
	Proportion	%	
Women <50 years old *(n = 22)*			
			
No risk reduction	11/12	92	
Risk reduction	1/10	10	0.0003
*BRCA*^-^	10/12	83	
*BRCA*^+^	2/10	20	0.008
*BRCA*^- ^women (*n *= 12)			
No risk reduction	9/9	100	
Risk reduction	1/3	33	0.045
*BRCA*^+ ^women (*n *= 10)			
No risk reduction	2/3	66	
Risk reduction	0/7	0	0.07
Women ≥50 years old (*n *= 21)			
			
No risk reduction	6/7	86	
Risk reduction	4/14	29	0.02
*BRCA*^-^	9/14	64	
*BRCA*^+^	1/7	14	0.06
*BRCA*^- ^women (*n *= 14)			
No risk reduction	6/6	100	
Risk reduction	3/8	38	0.03
*BRCA*^+ ^women (*n *= 7)			
No risk reduction	0/1	0	
Risk reduction	1/6	17	1.000

NAF, nipple aspirate fluid.
